# Homeopathic Individualized Q-Potencies versus Fluoxetine for Moderate to Severe Depression: Double-Blind, Randomized Non-Inferiority Trial

**DOI:** 10.1093/ecam/nep114

**Published:** 2011-06-08

**Authors:** U. C. Adler, N. M. P. Paiva, A. T. Cesar, M. S. Adler, A. Molina, A. E. Padula, H. M. Calil

**Affiliations:** Faculdade de Medicina de Jundiaí, Homeopathy Graduation Programme, Department of Psychobiology, Universidade Federal de São Paulo, R. Napoleão de Barros, 925 São Paulo, SP 04024-002, Brazil

## Abstract

Homeopathy is a complementary and integrative medicine used in depression, The aim of this study is to investigate the non-inferiority and tolerability of individualized homeopathic medicines [Quinquagintamillesmial (Q-potencies)] in acute depression, using fluoxetine as active control. Ninety-one outpatients with moderate to severe depression were assigned to receive an individualized homeopathic medicine or fluoxetine 20 mg day^−1^ (up to 40 mg day^−1^) in a prospective, randomized, double-blind double-dummy 8-week, single-center trial. Primary efficacy measure was the analysis of the mean change in the Montgomery & Åsberg Depression Rating Scale (MADRS) depression scores, using a non-inferiority test with margin of 1.45. Secondary efficacy outcomes were response and remission rates. Tolerability was assessed with the side effect rating scale of the Scandinavian Society of Psychopharmacology. Mean MADRS scores differences were not significant at the 4th (*P* = .654) and 8th weeks (*P* = .965) of treatment. Non-inferiority of homeopathy was indicated because the upper limit of the confidence interval (CI) for mean difference in MADRS change was less than the non-inferiority margin: mean differences (homeopathy-fluoxetine) were −3.04 (95% CI −6.95, 0.86) and −2.4 (95% CI −6.05, 0.77) at 4th and 8th week, respectively. There were no significant differences between the percentages of response or remission rates in both groups. Tolerability: there were no significant differences between the side effects rates, although a higher percentage of patients treated with fluoxetine reported troublesome side effects and there was a trend toward greater treatment interruption for adverse effects in the fluoxetine group. This study illustrates the feasibility of randomized controlled double-blind trials of homeopathy in depression and indicates the non-inferiority of individualized homeopathic Q-potencies as compared to fluoxetine in acute treatment of outpatients with moderate to severe depression.

## 1. Introduction

Depression was the most prevalent (19.2%) of the chronic diseases assessed by the Brazilian World Health Survey in 2003 [1], including asthma, arthritis, angina and diabetes.

There still remain flaws in the treatment of depression with antidepressants, in terms of efficacy, adverse effects, non-compliance to treatment and delayed onset of their therapeutic response [2–5]. Regarding efficacy, response has been defined as a decrease of 50% or more from baseline score in a rating scale, such as the Hamilton Rating Scale for Depression (HAM-D) or the Montgomery & Åsberg Depression Rating Scale (MADRS), whereas depression scores HAM-D ≤ 7 and MADRS ≤ 10 are often used to characterize remission [6]. Unmet needs of the conventional treatment may contribute to the patients' search for alternatives: depression is one of the leading causes for use of complementary and integrative medicine (CIM) in the USA [7], although any type of CIM has not yet conclusively had its efficacy demonstrated over placebo in that disease [8].

Homeopathy is an integrative medicine, also used in depression [9] and recognized as a medical specialty in Brazil. The classical homeopathy treatment is customized to the patient. The homeopathic medicine is individually selected according to the similitude to the patient's signs and symptoms, aiming at desensitizing the organism to the physical and mental alterations induced by disease. Minimal doses used in homeopathy are obtained by dynamization, the process developed by Hahnemann to prepare medicines through sequential agitated dilutions, in relatively small volumes [10]. Hahnemann's dynamization gained support of physics: thermoluminescence emitted by “ultra-high dilutions” (dynamizations) of lithium chloride and sodium chloride was specific of the salts initially dissolved, despite their dilution beyond the Avogadro number [11].

With homeopathic dynamized medicines given in such “uncommonly small doses”, Hahnemann aimed at achieving “a rapid, gentle and permanent restoration of the health”, which seemed to him easier to achieve with his last dynamization method known as fifty-millesimal, or Quinquagintamillesimal (Q-potencies), once the medicine is diluted *∼*50 000 times at each step (potency) of the dynamizing process [10]. Hahnemann's instructions for the use and preparation of these potencies were part of a posthumous publication (the 6th edition of the Organon), unknown by the homeopathic community until the last decades [12, 13].

There is no controlled study of the homeopathic use of Q-potencies in depressive disorders and the overall evidence for the efficacy of homeopathy in depression has been limited due to lack of clinical trials of high quality [14, 15]. Nevertheless, Q-potencies have been recently tested in randomized, controlled studies showing therapeutic effects in fibromyalgia and attention deficit hyperactivity disorder as compared to placebo [16, 17]. We have reported a series of cases of depression treated with individualized Q-potencies, stressing the need of controlled studies [18]. The present study was a further step, aiming at investigating the non-inferiority and tolerability of individualized homeopathic Q-potencies in adults with acute depression, as compared to fluoxetine, in a prospective, randomized, double-blind, double-dummy parallel trial.

## 2. Methods

### 2.1. Patients

Patients referred to the outpatient clinic of Homeopathy and Depression of Jundiaí Medical School (Faculdade de Medicina de Jundiaí, São Paulo, Brazil), who met DSM-IV criteria for depression (single or recurrent episode) following a Structured Clinical Interview—SCID [19] were included in the study. Capacity and willingness to give informed consent and to comply with study procedures were also required.

Exclusion criteria were: psychosis, mania, hypomania or any other Axis I disorder except panic disorder, personality disorders, history of seizures, history of alcohol or drug abuse 1 year prior to the screening, antidepressant use up to 30 days before screening, pregnancy or lactation, age <  18 years, MADRS score <  15, recent suicide planning or attempts, although these are symptoms of depression, they are also standard exclusion criteria in depression clinical studies, including CAM trials in depression [20].

The 91 patients were consecutively recruited between February 2006 and September 2008.

### 2.2. Ethics

A written informed consent was obtained from each participant. The study was approved by the Ethic Committees at FMJ and UNIFESP.

### 2.3. Study Design, Blinding and Randomization

The study was a prospective, randomized, double-blind, double-dummy trial, with fluoxetine as active control. The double-dummy methodology was used once it was not possible to make the homeopathic medication (hydroalcoholic solutions of the medicinal globules) and the fluoxetine capsules to look the same, so we created a placebo for each medicine.

Following inclusion, patients went through a homeopathic anamnesis with the principal investigator (U.C.A.) and received a prescription of the individualized homeopathic medicine and fluoxetine. The research pharmacist randomly delivered homeopathy and placebo or fluoxetine and placebo, according to a randomized assignment sequence to either homeopathy or fluoxetine group, generated by http://www.randomizer.org/ and with the code, 1 or 2, chosen by the study's senior author (H.M.C.).

The randomization sequence (one set of 100 non-unique numbers, ranging from 1 to 2, unsorted) was recorded and sent to the research pharmacist at the start of the study. Only the senior author and the pharmacist had access to the code of the randomized sequence during the study. After each patient completed the 8-week trial (or in emergency interventions—clinical worsening, disturbing adverse effects) the pharmacist informed the PI if the individual patient was taking homeopathy or fluoxetine (and the matched placebo) without disclosing the code.

### 2.4. Study Medications

Subjects at baseline received one of the following medications:

- one drop of the prescribed Q-potency, three times a week (on Mondays, Wednesdays and Fridays), in the morning, before breakfast or,
- one hard white gelatine capsule containing 20 mg fluoxetine-hydrochloride daily, in the morning, after breakfast.
- plus their matching placebos. A double-dummy technique with matching placebos for each active treatment was applied, thus both placebos seemed identical to their corresponding verum formulations.

Homeopathic Q-potencies were provided by HN-Cristiano Pharmacy, Pinheiros, São Paulo, under the responsibility of a pharmacist (Cesar, AT). They were supplied in 30 mL bottles, with one globule of the indicated Q-potency dissolved in 20 mL of a 30% alcohol-distilled water solvent. Patients began the study on Q2 potency and moved on to higher potencies in order: Q3, Q4, and so forth, according to medical indications. Placebo bottles were filled with the same amount of 30% alcohol.

Capsules of fluoxetine were provided by the High Cost Pharmacy of Jundiai's public health system, under the responsibility of a pharmacist (Luciana Teixeira Lencioni Lovate). As the capsules available at the local public health system came in yellow-green color, they were re-encapsulated in white color by another pharmacist (Regina Oliveira), at Pharmaessência Pharmacy, Campinas, SP, to match placebo white capsules containing celluloses, kaolin and talcum powder.

Both treatments were conducted as if the participants were receiving active treatment. In case of no response after 4 weeks of treatment, the patient blindly received:

- 40 mg of fluoxetine daily (20 mg b.i.d.) or two placebo capsules and
- a changed homeopathic prescription, or placebo solution. The homeopath was allowed to change remedy, potency or posology prescriptions.

The homeopath has a medical degree and 20 years of experience with the methodology described by Hahnemann in 6th edition of the Organon [21].

### 2.5. Measures

Improvement was measured by the MADRS, applied by a collaborator blind to treatment groups or outcomes. The MADRS scale has been chosen because it has been validated in Brazil and based on evidence that this instrument most accurately reflects treatment induced change [22–24].

The primary efficacy measure was mean change in the MADRS scores from baseline to the 4th and 8th weeks of treatment, whereas the secondary efficacy outcomes were response and remission rates at the same intervals.

Tolerability was assessed with the side effect rating scale of the Scandinavian Society of Psychopharmacology [25], applied by a collaborator blind to treatment groups or outcomes.

### 2.6. Statistics

The demographic characteristics and duration of illness were compared with Student's *t*-test for independent samples. Fisher's exact test was used for comparison of marital status and analysis of dropouts between the two groups.

A prefixed margin of non-inferiority (Δ) of 1.45 was specified, according to recommendation that Δ should be between one-third and one-half of the advantage of the active comparator over placebo and correspond with minimum difference that would be considered clinically important [26]. The margin of non-inferiority was assumed based on the mean MADRS-score changes of the placebo arm, from a multicenter placebo-controlled clinical study of moderate to severe depression [27]. The non-inferiority analysis included all 91 randomized patients, using a “full analysis set” [28], that is, with all observed MADRS scores, but without filling in the missing data. Non-inferiority of homeopathic individualized medicines over fluoxetine was accepted in a 0.025 level test, if the upper limit of the 95% confidence interval (CI) around the difference of the primary efficacy measures was situated below the limit of non-inferiority.

Analysis of the MADRS scores follow-up was made with repeated measures analysis of variance (ANOVA), with time as within factor and condition as between factor, and Bonferroni's multiple comparisons method. Response and remission rates were analyzed with non-parametric analysis for longitudinal data. Sample size was not calculated because this trial was a sequence of a pilot study, with a smaller sample (*n* = 59), but already sufficient to suggest the non-inferiority of homeopathy to fluoxetine.

## 3. Results

**Figure 1:**
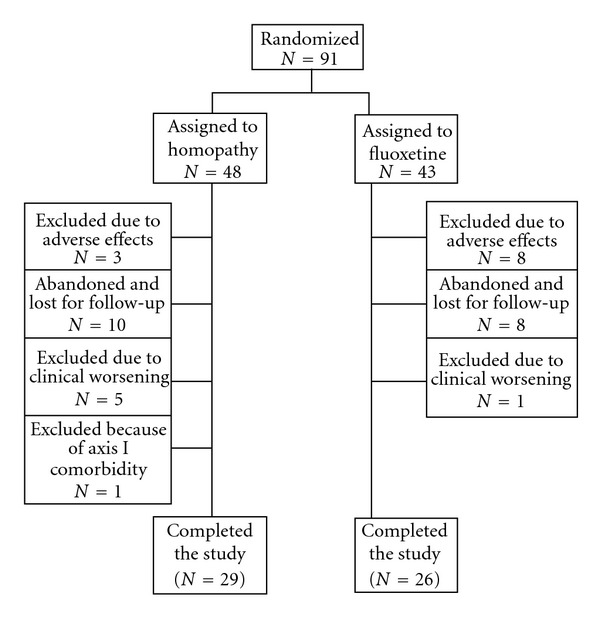
Diagram flow of subjects throughout the study.

This sample consisted of patients with moderate to severe depression, because their mean MADRS depression scores were close to the 31 score cut-off for moderate and severe depression [29]. Initially, 284 subjects were screened, 105 of them met the inclusion criteria, 14 out of them did not attend the first appointment, 91 were randomized and 55 completed the 8-week trial. A detailed flow chart of subject progress through the study is shown in Figure 1.

There were no significant differences between the proportions of excluded and lost for follow-up patients in the two groups (*P* = .99), though there was a trend toward greater treatment interruption for adverse effects in the fluoxetine group, as can be seen in Table 1.

Almost all patients enrolled in the study were female: 89/91 (98%). One male patient was randomly assigned to each group. There was no significant difference in the marital status (married, single, widow, divorced) between the two groups (*P* = .86). Other baseline characteristics were also similar in the fluoxetine and homeopathy groups, as shown in Table 2.

Twenty medicines were used to treat the 48 patients randomized to homeopathy: *Alumina*, *Anacardium orientale*, *Arsenicum album*, *Aurum foliatum*, *Baryta carbonica*, *Calcarea carbonica*, *Carbo animalis*, *Causticum*, *Graphites*, *Hepar sulphuris calcareum*, *Kali carbonicum*, *Lycopodium clavatum*, *Natrum carbonicum*, *Natrum muriaticum*, *Mezereum*, *Phosphorus*, *Sepia succus*, *Silicea terra*, *Sulphur* and *Zincum*. These medicines were selected according to Hahnemann's instructions, that is, matching the characteristic symptoms (the stronger, well-marked and peculiar symptoms) of each case to very similar symptoms described by healthy volunteers in homeopathic drug trials [21].

Regarding concomitant psychoactive medications, in the fluoxetine group three patients were taking clonazepam (1–2.5 mg) and two were on diazepam (5–10 mg). In the homeopathy group, one patient was using clonazepam and another one was on diazepam at the beginning of the study (same dosage range). No patient referred to this study was on psychotherapy.

### 3.1. Primary Efficacy Analysis

Repeated measures ANOVA were used with time as within factor and treatment condition as between factor. The results showed significant differences for time (within factor, *P* < .001), but not for treatment group (between factor, *P* = .105) interaction (*P* = .749).

**Figure 2:**
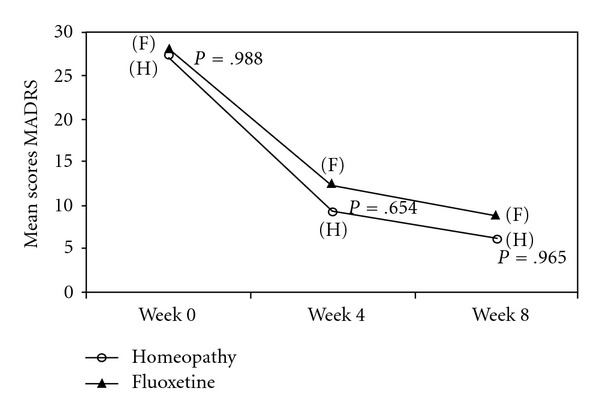
MADRS mean scores at baseline and on 4th and 8th weeks of randomized treatment with fluoxetine or individualized homeopathic Q-potencies (ITT population).

Both treatment groups started with similar depression mean scores: fluoxetine 28.09 ± 6.88 (*n* = 43), homeopathy 27.21 ± 6.22 (*n* = 48, *P* = .988) and improved during the 8 weeks of double-blind treatment. The statistical analysis showed that the differences between the MADRS scores in the two groups were not significant (as shown in Figure 2), neither at the 4th week—fluoxetine 12.33 ± 8.52 (*n* = 36), homeopathy 9.29 ± 8.31 (*n* = 38, *P* = .654) nor at the 8th week—fluoxetine 8.85 ± 7.48 (*n* = 26), homeopathy 6.21 ± 4.99 (*n* = 29, *P* = .965).

**Figure 3:**
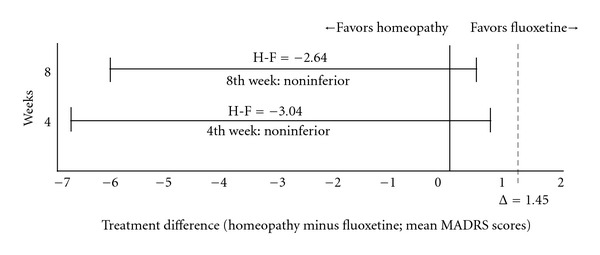
Non-inferiority representation of the difference (homeopathy versus fluoxetine) in the mean change of the MADRS scores on the 4th and 8th weeks of randomized, double-bind treatment. Error bars indicate two-sided 95% CIs. Tinted area indicates zone of non-inferiority. Delta indicates the margin of non-inferiority. Mean differences (homeopathy-fluoxetine) were −3.04 (−6.95 to 0.86) and −2.64 (−6.05 to 0.77) at weeks 4th and 8th, respectively.

In line with the MADRS mean changes illustrated in Figure 2, the non-inferiority analysis showed that the individualized homeopathic Q-potencies were not inferior to fluoxetine, once the upper limit of the CIs lies to the left of Δ and includes zero [28], as represented by Figure 3.

### 3.2. Secondary Efficacy Analysis

Fluoxetine and homeopathy demonstrated similar response rates on the 4th (63.9 and 65.8%, resp.) and 8th (84.6 and 82.8%, resp.) weeks of treatment. Also no significant differences were found for the remission rates, on the 4th (47.2 and 55.3%, resp., *P* = .422) and 8th (76.9 and 72.4%, resp., *P* = .716) weeks of treatment.

### 3.3. Tolerability

There were also no significant differences between the side effects rates, although a higher percentage of patients treated with fluoxetine (21.4%) than those who received homeopathy (10.7%) reported “side effects that interfere markedly with the patient's performance” [25] (*P* = .275).

## 4. Discussion

In this study, depressed outpatients were randomly assigned to a double-blind treatment with individualized homeopathic Q-potencies or fluoxetine. The non-inferiority analysis indicated that the homeopathic Q-potencies were not inferior as compared to fluoxetine in treatment of this sample of outpatients with moderate to severe depression.

This is the first randomized controlled double-blind trial with a reasonable number of subjects to draw conclusions about the homeopathic treatment of depression, to the best of our knowledge. In fact, a recent systematic review found only two randomized controlled trials examining the use of homeopathy to treat depression, one of low methodological quality (non-blinded) and the other with recruitment‘s difficulties: eleven participants were included and only three completed the study [30–32].

The current sample was not recruited by advertisement and it was not composed by “consumers of alternative medicine” [33], but by help-seeking patients referred to clinic of Homeopathy and Depression of Jundiaí Medical School by health care professionals within the public health system. The predominance of women participants in a proportion greater than normally expected may be partially explained by men's relatively limited use of public health services in Brazil, a trend that has been associated with representation of caring as a female task, work-related issues, difficult access to services and lack of services specifically targeting men's health [34].

The need of individual prescriptions in classical homeopathy has been considered as “a severe obstacle for any double-blind trial” by experienced researchers [17]. In fact, a study design in which the selection of a suitable, individualized homeopathic medicine occurs during the double-blind randomized phase evaluates not only the efficacy of homeopathy, but also the efficiency of the homeopath in selecting and managing that medicine. A placebo substitution design (with an open-label phase preceding the randomization) would be recommendable, but in depression studies such a design is used for continuation or maintenance trials [35] and not to assess the treatment of the acute episode.

Primary efficacy measure results indicated mean MADRS scores differences were neither significant at the 4th week (*P* = .654), nor at the 8th week (*P* = .965). There were also no significant differences between response or remission rates in the two treatment groups, which were over 70% and in some degree superior to those found in primary care settings for active antidepressant interventions, favoring the hypothesis that “the homeopathic consultation is in itself a therapeutic intervention working independently or synergistically with the prescribed remedy” [36].

A placebo-arm was not included in the present study because it was not authorized by the National Ethic Council. Although placebo interventions are associated with mean response or remission rates of *∼*35% [37, 38], a placebo effect cannot be ruled out, since the homeopathic Q-potencies were compared with an antidepressant and “it is becoming more and more difficult to prove that antidepressants—even well-established antidepressants—actually work better than placebo in clinical trials” [39]. Nevertheless, it also has to be taken into consideration that the antidepressant-placebo difference seems to be smaller in the trials aiming at mild to moderate depression [40, 41] and the present sample consisted of patients suffering from moderate to severe depression. Placebo-controlled studies would be recommendable to clarify these findings.

Fluoxetine and homeopathy patients showed differences, although not significant, in exclusion profiles and tolerability. There was trend toward greater treatment interruption for adverse effects in the fluoxetine group, what is in line with the higher percentage of troublesome adverse effects reported by patients receiving fluoxetine. On the other hand, more patients randomized to homeopathy than to fluoxetine were excluded due to worsening of their depressive symptoms. Possible explanations are that casual differences can occur in small samples, or that homeopathy was not effective in protecting against stressful situations or even that the medicines selected were non-homeopathic, that is, not adequately individualized to match the peculiar symptoms of each case. There is no data about the efficacy of homeopathy in protecting against depression relapse or recurrence, but it's known that stressful life events can cause recurrence of depression even in conventionally medicated patients [42].

The current study has other limitations besides the lack of a placebo control, such as dependence on a single homeopathic practitioner, a relatively small sample and a short period of treatment—the acute phase of depression. A multicenter trial could include a larger number of participants, from different homeopathic research centers, increasing the generalizability of the results. Nevertheless, larger or multicenter trials aiming at repeating these results should take in account the need for properly training the physicians in the homeopathic methodology used (6th edition of the Organon), as well as the use of high quality, exactly prepared Q-potencies.

A recent meta-analysis of homeopathic trials concluded that the results were “compatible with the notion that clinical effects of homeopathy are placebo effects” [43]. However, as demonstrated by Lüdtke and Rutten, this conclusion was based on an arbitrarily chosen subset of eight trials, out of 21 high-quality trials and the results favor homeopathy, if another threshold to define a “large trial” is used [44]. Moreover, the homeopathic interventions were grouped in classical, clinical, complex or isopathy, without any further reference to the specific homeopathic clinical or pharmaceutical methodology used in each one of these groups. Defining the homeopathic methodology being analyzed would have been essential to avoid biased or generalized conclusions. In an analogous way, the efficacy of psychotherapeutic interventions in depression is assessed within their specific approaches: behavioral, cognitive-behavior, interpersonal, and so forth, [45].

This study, in spite of its limitations, illustrates the feasibility of randomized controlled double-blind trials of homeopathy for depression and indicates the non-inferiority of individualized homeopathic Q-potencies as compared to fluoxetine in the acute treatment of outpatients with moderate to severe depression. Further studies are needed to confirm these results, as well as studies aiming at the continuation and maintenance phases of depression treatment with homeopathy.

## Figures and Tables

**Table 1:** Excluded or lost for follow-up patients.

Discontinuance reasons	Homeopathy *n* (%)	Fluoxetine *n* (%)	*P*	Test
Adverse effects	3 (6.3)	8 (18.6)	.071	Chi-square test
Lost for follow-up	10 (20.8)	8 (18.6)	.79	Chi-square test
Worsening	5 (10.4)	1 (2.3)	.207	Fisher's exact test
Comorbidity	1 (2.1)^a^	0	1.00	Fisher's exact test

^a^Bulimia Nervosa.

**Table 2:** Baseline demographic and clinical characteristics.

Baseline parameters	Fluoxetine,		Homeopathy,		*P*-values
	*N* = 43		*N* = 48		
	Mean	±SD	Mean	±SD
Age (years)	41.9	12.3	44.3	11.8	.345
Offspring (number of children)	1.9	1.4	2.2	1.3	.229
School background (years)	8.0	4.2	7.4	3.6	.471
Duration of illness (years)	4.8	7.4	4.6	7.8	.883
MADRS scores	28.1	6.9	27.2	6.2	.523
